# Cattle grow taller: Implications of outdated ordinal scores for genetic evaluations and selection?

**DOI:** 10.3168/jdsc.2025-0930

**Published:** 2026-01-30

**Authors:** Xiao-Lin Wu, Allicia Horn, Ezequiel Nicolazzi, John B. Cole

**Affiliations:** 1 Council on Dairy Cattle Breeding, Bowie, MD 20716; 2 Department of Animal and Dairy Sciences, University of Wisconsin–Madison, Madison, WI 53706; 3 Brown Swiss Association, Beloit, WI 53511; 4 Department of Animal Sciences, Donald Henry Barron Reproductive and Perinatal Biology Research Program, and the Genetics Institute, University of Florida, Gainesville, FL 32611; 5 Department of Animal Science, North Carolina State University, Raleigh, NC 27607; 6 Department of Animal Biosciences, University of Guelph, Ontario, Canada N1G 2W1

## Abstract

In dairy cattle, conformation and linear-type traits are routinely recorded as categorical or ordinal phenotypes. Some traits originating from continuous biological measurements are discretized into categorical scores. These traits are moderately heritable and play a crucial role in breeding programs, serving as indicators of longevity, fertility, and overall lifetime productivity. However, over time, the underlying phenotypic distributions can evolve due to selection, management, and environmental changes. As a result, the mapping between biological measurements and categorical scores may become obsolete. This paper investigates phenotypic distribution shifts within ordinal scoring systems, using stature in Brown Swiss cattle as a case study. We present an analytical characterization of these shifts and their genetic consequences. A simulation study based on a real pedigree was conducted to quantify the extent of these effects and to explore methods for predicting these changes. The results emphasize periodic recalibration of threshold boundaries to keep the ordinal scoring systems aligned with current population distributions and safeguard the validity of genetic improvement programs. We anticipate that our findings will provide practical guidance for detecting, interpreting, and managing distributional shifts in categorical phenotypes within structured genetic evaluation and selection programs.

## Highlights

• Phenotypic distribution shifts are analytically examined and quantitatively characterized.
• Their impacts are revealed, with emphasis on genetic evaluations and selection accuracy.
• Outdated scores distort the genetic evaluations and compromise the selection outcomes.
• The results emphasize regular updates of binning thresholds to reflect population shifts.
• Best linear projection is introduced to predict and interpret phenotypic and genetic shifts.

## Graphical Abstract

**Figure:**
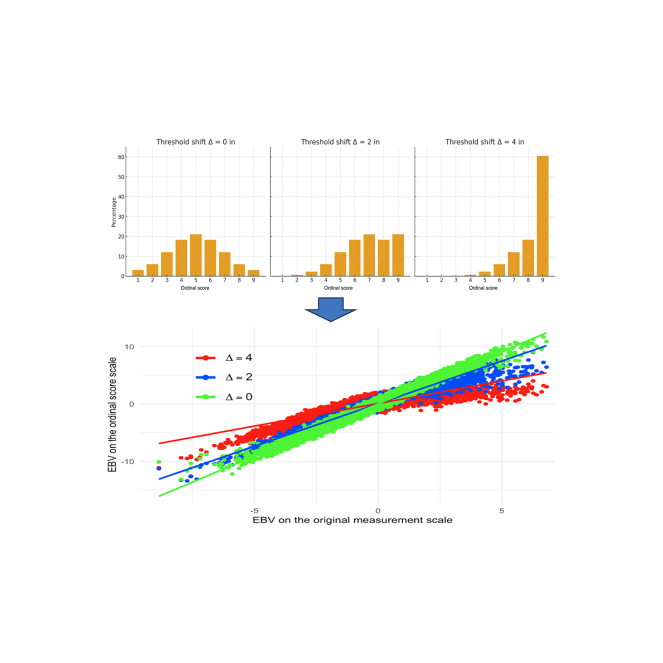
**Summary:** In dairy cattle, conformation and linear-type traits are routinely recorded as categorical or ordinal phenotypes than as continuous measurements. However, phenotypes can evolve over time due to selection, management, and environmental change. As a result, the mapping between underlying biological measurements and categorical scores may become outdated. This article examines phenotypic distribution shifts and their impacts on genetic evaluations and selection outcomes in an ordinal scoring system, using stature in US Brown Swiss cattle as a case study. Upper panel: illustration of shifts in the distribution of a 1 to 9 ordinal score under varied scenarios (Δ = 0, 2, 4 inches; 0, 5.08, 10.16 cm, respectively); lower panel: scatterplots comparing estimated breeding values (EBV) derived from the continuous phenotype scale with those derived from the ordinal score scale under the same 3 phenotype-shift scenarios (Δ = 0, 2, 4 inches).

In dairy breeding and management, ordinal (linear-type) scoring provides a practically convenient and standardized framework for describing morphological and structural traits across herds and environments, typically on a 1 to 9 scale, with some systems extending to 50 or even 80 categories for finer discrimination. These traits are generally moderately to highly heritable (Veerkamp and Brotherstone, 1997; Manafiazar et al., 2016), serving as indirect indicators of important functional traits such as longevity, fertility, and overall lifetime productivity (VanRaden and Wiggans, 1995).

Linear scores are assigned according to well-defined biological attributes or biometric features. However, some traits originate from continuously measured variables that are subsequently transformed into categorical scores. For example, stature is inherently continuous but is routinely converted into a 1–9 scale, with each unit representing a 1-inch (**in**) interval. In the US Brown Swiss Association (**BSA**; Beloit, WI), for instance, the intermediate score of 5 corresponds to a stature of 56 in (142.24 cm). Animals shorter than 56 in receive scores from 1 (52 in) to 4 (55 in), whereas taller animals are assigned scores from 6 (57 in) to 9 (60 in).

Over time, however, the phenotypic distributions of such traits can shift. For example, modern cattle have become taller (Visscher and Goddard, 2011), largely due to correlated selection responses. Because height was rarely a primary breeding target, long-term selection for milk yield, angularity, and overall type possibly led to consistent upward drift in frame size. Height (stature) is correlated with udder depth, which is in turn associated with improved udder health. Taller cows carry their udders higher off the ground, reducing the risk of intramammary infections from environmental pathogens. In a recent survey of 5,279 Brown Swiss cows in 2024, we observed a mean ± SD stature score of 6.65 ± 1.45, indicating a clear upward shift in the underlying stature measurements, with the central tendency moving from the intended midpoint of 5 to approximately 7 on the ordinal scale. To address this discrepancy, the BSA announced a recalibration of the scoring system, raising all stature thresholds by 2 in, effective in the April 2025 genetic evaluation. This realignment of stature scores reflects current biological reality; however, the broader implications of such shifts in phenotypic distributions have not yet been systematically evaluated.

When categorical scaling systems are incorporated into genetic evaluations, key questions arise: How do phenotypic shifts in ordinal scores affect EBVs and heritability estimates? Furthermore, what are the genetic and selection consequences of re-anchoring the scoring scale? Therefore, the objectives of this study were to investigate phenotypic distribution shifts in ordinal scoring systems, using stature in US Brown Swiss (**BS**) cattle as a case study. We present an analytical framework to characterize these shifts and evaluate their consequences on phenotypic distributions, genetic evaluations, and selection outcomes.

First, how do ordinal scores change under a shifting phenotypic distribution? Let **y** = {*y_i_*|*i* = 1, . . ., *n*} denote a vector of continuous trait values for *n* animals, which are later discretized into ordinal scores using the equal-interval (**EI**) binning method, the standard approach adopted by the BSA. Briefly, the EI approach divides the range of continuous phenotypic values into *k* ordered categories (bins) of equal width, with the first and last bins defined as open-ended intervals to accommodate extreme observations. The thresholds are defined as

$$-\infty =\tau_{0}<\tau_{1}<...<\tau_{k-1}<\tau_{k}=+\infty .$$

Here, *τ_j_*_−1_ and *τ_j_* define the lower and upper boundaries for the *j*th bin.

For individual *i*, an ordinal score (*s_i_*) is assigned as follows:

*s_i_* = *j* if *τ_j_*_−1_ ≤ *y_i_* < *τ_j_*, for *j* = 1, . . ., *k*.

$$\mathrm{Assume}y_{i}\&sim;N\left(\mu ,\sigma_{e}^{2}\right),$$

where *µ* is the population mean and $\sigma_{e}^{2}$ is the variance. The unconditional probability (**Pr**) of an observation falling into bin *j* is

[1] $$\begin{array}{c} p_{j}:=\mathrm{Pr}\left(s_{i}=j\right)=\mathrm{Pr}\left(\tau_{j-1}\leq y_{i}<\tau_{j}\right)\\ =\Phi \left(\frac{\tau_{j}-\mu }{\sigma_{e}}\right)-\Phi \left(\frac{\tau_{j-1}-\mu }{\sigma_{e}}\right), \end{array}$$

where $\Phi \left(\cdot \right)$ denotes the cumulative distribution function of the standard normal distribution. The mean and variance of the ordinal score (*s*) are then given by

[2] $$E\left(s\right)=\sum_{j=1}^{k}jp_{j};$$

[3] $$\mathrm{Var}\left(s\right)=\sum_{j=1}^{k}j^{2}p_{j}-{\left(E\left(s\right)\right)}^{2}.$$

For instance, let the current population mean be *µ* = 58 in (147.32 cm) with SD of *σ_y_* = 1.87 in (4.75 cm). The 9 categories are defined using a fixed 1-in interval for all bins except the first and the last categories: 1 (*y* < 54.5 in), 2 (54.5 ≤ *y* < 55.5 in), 3 (55.5 ≤ *y* < 56.5 in), 4 (56.5 ≤ *y* < 57.5 in), 5 (57.5 ≤ *y* < 58.5 in), 6 (58.5 ≤ *y* < 59.5 in), 7 (59.5 ≤ *y* < 60.5 in), 8 (60.5 ≤ *y* < 61.5 in), and 9 (*y* ≥ 61.5 in). Here, the US customary units are retained for consistency with industry reporting. Under *y* ∼ *N*(58, 1.87^2^), the proportions of observations falling in each group are approximately 3.06% (groups 1 or 9), 5.95% (groups 2 or 8), 12.2% (groups 3 or 7), 18.2% (groups 4 or 6), and 21.2% (group 5). These proportions are symmetric around the central category (group 5) and sum to 100% (see the top-left panel of the Graphical Abstract).

Now, suppose the current scores resulted from shifting upward from the outdated scores (denoted by $s_{i}^{*})$ by Δ = 2 in (5.08 cm). Equivalently, this corresponds to shifting all interior thresholds downward by Δ = 2 in (5.08 cm) while keeping the current continuous phenotypic values fixed. The probability of an observation falling into the *j*th category under the outdated scheme is

[4] $$p_{j}^{*}:=\mathrm{Pr}\left(s_{j}^{*}=j\right)=\Phi \left(\frac{\tau_{j}-\mu -\Delta }{\sigma_{e}}\right)-\Phi \left(\frac{\tau_{j-1}-\mu -\Delta }{\sigma_{e}}\right).$$

Applying these outdated thresholds yields the following proportions of observations across the 9 categories: 0.16% (group 1), 0.64% (group 2), 2.26% (group 3), 5.95% (group 4), 12.2% (group 5), 18.2% (group 6), 21.2% (group 7), 18.2% (group 8), and 21.2% (group 9). The downward-shifted thresholds thus lead to a disproportionate accumulation of observations in higher score groups (7–9), whereas lower groups (1–3) become sparsely populated (see the top-middle panel of the Graphical Abstract).

When the thresholds are further downscaled by Δ = 4 in (10.16 cm), the proportions of observations across the 9 categories become 0.003%, 0.023%, 0.134%, 0.64%, 2.26%, 5.95%, 12.2%, 18.2%, and 60.6%, respectively. In this case, nearly two-thirds of the population is clustered in the highest category, while the lower categories become almost empty (see the top-right panel of the Graphical Abstract).

Failing to recalibrate the scoring system periodically leads to severe misalignment between ordinal scores and the underlying continuous phenotypes. The squared (and rank) correlations between the ordinal scores and the true continuous phenotypes decrease from 0.969–0.970 (0.987) for Δ = 0 (in) to 0.924–0.926 (0.983) for Δ = 2 (in), and then to 0.697–0.700 (0.879) for Δ = 4 (in). The sharp decline in both metrics demonstrates a substantial loss of information and deterioration in phenotypic ranking accuracy as scoring thresholds become outdated.

Secondly, how do phenotypic shifts influence genetic evaluations? Consider the following linear animal model for the latent continuous phenotype (***y***):

[5] **y** = **1***µ* + **u** + **e**.

Here, **1** represents a vector of ones and $u\&sim;N\left(0,A\sigma_{u}^{2}\right)$ is a vector of additive genetic effects, where **A** is the numerator additive genetic relationship matrix and $\sigma_{u}^{2}$ is the additive genetic variance. The residual vector $e\&sim;N\left(0,I\sigma_{e}^{2}\right),$ with $\sigma_{e}^{2}$ denoting the residual variance and **I** the identity matrix. The vectors **u** and **e** are assumed to be mutually independent.

When fitting the same linear animal model to the ordinal scores **s**, we write the following:

[6] **s** = 1*µ*_s_ + **u***_s_* + **e***_s_*,

where $u_{s}\&sim;N\left(0,A\sigma_{u_{s}}^{2}\right)$ and $e_{s}\&sim;N\left(0,I\sigma_{e_{s}}^{2}\right),$ with **u***_s_* and **e***_s_* mutually independent.

The link between the latent-scale *u* and the score-scale *s* can be quantified analytically through the following best linear projection (**BLP**):

[7] *s_i_* = *α* + *βu_i_* + *r_i_*,

where *α* and *β* are the intercept and regression coefficient, respectively, and *r_i_* is a residual term with E(*r_i_*) = 0 and Cov(*r_i,_u_i_*) = 0. The BLP slope is defined by

[8] $$\beta =\frac{C\mathrm{ov}\left(s_{i},u_{i}\right)}{\mathrm{Var}\left(u_{i}\right)}.$$

Define the conditional mean function:

$$\begin{array}{c} g\left(u_{i}\right):=E\left(s_{i}|u_{i}\right)=\sum_{j=1}^{k}j\mathrm{Pr}\left(s_{i}=j|u_{i}\right)\\ =k-\sum_{j=1}^{k-1}\Phi \left(\frac{\tau_{j}-\mu -u_{i}}{\sigma_{e}}\right), \end{array}$$

where *g*(*u_i_*) is the conditional expectation of the ordinal score given the breeding value. Because *s_i_* depends on *u_i_* only through $g\left(u_{i}\right)=E\left(s_{i}|u_{i}\right),$ we have

$$\mathrm{Cov}\left(s_{i},u_{i}\right)=\mathrm{Cov}\left(g\left(u_{i}\right),u_{i}\right).$$

Stein's lemma implies (Stein, 1956; Casella and Berger, 2002) that, valid for $u_{i}\&sim;N\left(0,\sigma_{u}^{2}\right),$

$$\mathrm{Cov}\left(g\left(u_{i}\right),u_{i}\right)=\sigma_{u}^{2}E\left(g^{'}\left(u_{i}\right)\right),$$

and, therefore,

$$\beta =\frac{\sigma_{u}^{2}E\left(g^{'}\left(u_{i}\right)\right)}{\sigma_{u}^{2}}=E\left(g^{'}\left(u_{i}\right)\right).$$

Differentiating *g*(*u_i_*) with respect to *u_i_* gives

$$g^{'}\left(u_{i}\right)=\frac{1}{\sigma_{e}}\sum_{j=1}^{k-1}\phi \left(\frac{\tau_{j}-\mu -u_{i}}{\sigma_{e}}\right),$$

where $\phi \left(\cdot \right)$ is the standard normal probability density function (**PDF**).

Taking expectation over $u_{i}\&sim;N\left(0,\sigma_{u}^{2}\right)$ yields the Gaussian convolution:

[9] $$\beta =E\left(g^{'}\left(u_{i}\right)\right)=\frac{1}{\sigma_{y}}\sum_{j=1}^{k-1}\phi \left(\frac{\tau_{j}-\mu }{\sigma_{y}}\right),\sigma_{y}^{2}=\sigma_{e}^{2}+\sigma_{u}^{2}.$$

Intuitively, when averaging a Gaussian PDF evaluated at linearly shifted arguments over a Gaussian random shift *u*, averaging “smooths” the PDF by convolving the 2 normals, which simply adds their variances: $\sigma_{y}^{2}=\sigma_{e}^{2}+\sigma_{u}^{2},$ and the prefactor 1/*σ_y_* is the new normalizing scale.

The BLP [7] induces a natural projection-based variance decomposition on the score scale:

[10] $$\mathrm{Var}\left(s_{i}\right)=\mathrm{Var}\left(E\left(s_{i}|u_{i}\right)\right)+E\left(\mathrm{Var}\left(s_{i}|u_{i}\right)\right)\approx \beta^{2}\sigma_{u}^{2}+\sigma_{r}^{2},$$

where $\sigma_{r}^{2}=Var\left(r_{i}\right).$ Define the BLP-implied additive genetic variance and BLP-implied residual variance on the ordinal scale as

[11] $$\sigma_{u_{s}}^{2}:=\mathrm{Var}\left(g\left(u_{i}\right)\right)\approx \mathrm{Var}\left(\alpha +\beta u_{i}\right)=\beta^{2}\mathrm{Var}\left(u_{i}\right)=\beta^{2}\sigma_{u}^{2};$$

[12] $$\sigma_{e_{s}}^{2}:=E\left(\mathrm{Var}\left(s_{i}|u_{i}\right)\right)\approx \mathrm{Var}\left(s_{i}\right)-\beta^{2}\sigma_{u}^{2}.$$

Hence, the projection-based heritability on the ordinal scale is

[13] $$h_{s}^{2}:=\frac{\sigma_{u_{s}}^{2}}{\sigma_{u_{s}}^{2}+\sigma_{e_{s}}^{2}}\approx \frac{\beta^{2}\sigma_{u}^{2}}{\mathrm{Var}\left(s\right)}.$$

The preceding approximation mappings depend on *µ*, $\sigma_{u}^{2},$ $\sigma_{e}^{2},$ and $\left(\tau_{j}\right),$ but not on the structure of **A**. Thus, they provide a direct analytical approach to predict how phenotypic shifts rescale the score-breeding value relationship through *β*. However, the EBV precisions (e.g., their prediction error variances) still depend on **A** and the amount of information. These approximations are good as long as the score has many categories (*k* is not too small) and thresholds are not too extreme.

Although these approximations are used in the present study, the exact definitions are the following:

$$\mathrm{Var}\left(g\left(u_{i}\right)\right)=E[g\left({u_{i})}^{2}\right)-{\left(E\left(g\left(u_{i}\right)\right)\right)}^{2},$$

where both expectations are one-dimensional Gaussian integrals, typically computed by Gauss–Hermite quadrature or Monte Carlo. Likewise,

$$\mathrm{Var}\left(s_{i}|u_{i}=u\right)=E\left(s_{i}^{2}|u_{i}=u\right)-g{\left(u\right)}^{2},$$

where $E\left(s_{i}^{2}|u_{i}=u\right)=\sum_{j=1}^{k}j^{2}\;\mathrm{Pr}\left(s_{i}=j|u_{i}=u\right)$ and $\mathrm{Pr}\left(s_{i}=j|u_{i}=u\right)=\Phi \left(\frac{\tau_{j}-\mu -u}{\sigma_{e}}\right)-\Phi \left(\frac{\tau_{j-1}-\mu -u}{\sigma_{e}}\right).$

Now, consider downscaling the threshold by Δ > 0. Employing the same BLP of $s_{i}^{*}$ on *u_i_*, the slope becomes

[14] $$\beta^{*}=\frac{1}{\sigma_{y}}\sum_{j=1}^{k-1}\phi \left(\frac{\tau_{j}-\mu -\Delta }{\sigma_{y}}\right).$$

Similar to the expressions [11] to [13], the projected variances and heritability under the downscaled thresholds are

[15] $$\sigma_{u_{s}}^{*2}\approx {\left(\beta^{*}\right)}^{2}\sigma_{u}^{2};$$

[16] $$\sigma_{e_{s}}^{*2}\approx \mathrm{Var}\left(s^{*}\right)-{\left(\beta^{*}\right)}^{2}\sigma_{u}^{2};$$

[17] $$h_{s}^{*2}\approx \frac{{\left(\beta^{*}\right)}^{2}\sigma_{u}^{2}}{\mathrm{Var}\left(s^{*}\right)}.$$

The mean and variance of the ordinal scores under the downscaled thresholds are $E\left(s^{*}\right)$ = $\sum_{j=1}^{k}jp_{j}^{*}$ and $\mathrm{Var}\left(s^{*}\right)$ = $\sum_{j=1}^{k}j^{2}p_{j}^{*}$ − ${\left(E\left(s^{*}\right)\right)}^{2}.$

As Δ increases, probability mass accumulates in the uppermost category (ceiling effect), and both β* and Var(s*) decrease toward zero, leading to smaller score-scale heritability and reduced EBVs.

Rescaling the ordinal scores by a constant *c* changes variance components but not the heritability estimates. Let **l** = *c***s**. The model becomes

[18] **l** = 1*µ_l_* + **u***_l_* + **e***_l_*,

where *µ_l_*, **u***_l_*, and **e***_l_* denote the overall mean, additive genetic, and residual effects on the rescaled ordinal scale. The rescaled variances are

[19] $$\mathrm{Va}r\left(l\right)=c^{2}\mathrm{Var}\left(s\right);$$

[20] $$\sigma_{u_{l}}^{2}\approx c^{2}\beta^{2}\sigma_{u}^{2}=c^{2}\sigma_{u_{s}}^{2};$$

[21] $$\sigma_{e_{l}}^{2}\approx c^{2}\left(\mathrm{Var}\left(s\right)-\sigma_{u_{s}}^{2}\right)=c^{2}\sigma_{e_{s}}^{2}.$$

However, heritabilities remain invariant under rescaling:

[22] $$h_{l}^{2}=\frac{c^{2}\sigma_{u_{s}}^{2}}{c^{2}\sigma_{u_{s}}^{2}+c^{2}\sigma_{e_{s}}^{2}}=\frac{\sigma_{u_{s}}^{2}}{\sigma_{u_{s}}^{2}+\sigma_{e_{s}}^{2}}=h_{s}^{2}.$$

A simulation study was conducted to quantify the effects of phenotypic distribution shifts on genetic evaluations. Simulated stature records were generated for 5,141 animals, drawn from a subset of BS cattle based on a real pedigree containing 12,909 unique individuals. The pedigree was truncated to 3 generations. Two alternative population means were used: 147.32 cm (58 in) and 142.24 cm (56 in). An SD of 4.75 cm (1.87 in) was applied, ensuring a binning width comparable to that used by the BSA. The mean of 147.32 cm represents the newly updated BSA stature scoring system (denoted as EI_1), whereas 142.24 cm represents the mean of the previous scoring system (denoted as EI_0). For simplicity, the overall mean was treated as the sole fixed effect, whereas the random effects included additive genetic values and residuals. Heritability was set to 0.43, as reported by Wiggans et al. (2004).

The 1–9 scores were subsequently rescaled to a 1–50 range by multiplying by *c* = 5, consistent with the evaluation scale used by the Council on Dairy Cattle Breeding (CDCB, USA) for BS cattle. Genetic evaluations were performed using an animal model under various scenarios—M0 (the baseline model): the continuous stature measurements as phenotypes; M1A (Δ = 0): group means on the continuous scale as phenotypes; M1B (Δ = 0): 5× 1–9 scores derived without distribution shifts as phenotypes; M2 (Δ = 2): 5× 1–9 scores with thresholds downscaled by Δ = 2 in as phenotypes; M3 (Δ = 4): 5× 1–9 scores with thresholds downscaled by Δ = 4 in as phenotypes. Accordingly, models M2 and M3 represent the old ordinal scoring systems, which are ineffectively updated (Δ = 2) or not updated at all (Δ = 4).

Model parameters were estimated using Bayesian inference via Markov chain Monte Carlo, assuming a flat prior for the overall mean and scaled inverse chi-squared priors for genetic and residual variances (Sorensen and Gianola, 2002). To minimize Monte Carlo error, each model was replicated 10 times, and the posterior means were averaged across replicates.

The baseline model M0 yielded a heritability estimate of h^2^ = 0.433 (Table 1), closely matching the simulated value (0.43). When the ordinal scores were generated using thresholds consistent with the current phenotypic distribution, the estimated heritability (h^2^ = 0.420–0.421) remained roughly consistent with the simulated heritability, regardless of whether the population means or ordinal scores were used as the phenotypes. However, when the scoring system was incorrectly or not updated, as in the cases of M2B (Δ = 2) and M3B (Δ = 4), the analyses produced systematically underestimated variance components compared with those from M1B (Table 1). In relative terms, the estimated genetic variance contracted more sharply than the estimated residual variance, reducing heritability to h^2^ = 0.409 (M2B) and h^2^ = 0.339 (M3B).

**Table 1:** Variance components, heritability estimates, and rank correlations obtained under various scenarios; numbers inside parentheses are SD across 10 replicates^1^, ^2^, ^3^

Model	$\sigma_{y}^{2}$	$\sigma_{u}^{2}$	$\sigma_{e}^{2}$	$S_{u}^{2}$	$S_{e}^{2}$	$h^{2}$	$\rho_{y}$	$\rho_{u}$
M0	22.7 (0.250)	10.0 (0.846)	13.1 (0.708)	4.91 (0.620)	8.42 (0.826)	0.433 (0.033)	1 (0)	0.717 (0.008)
M1A: Δ = 0	22.0 (0.254)	9.44 (0.951)	13.0 (0.798)	4.42 (0.728)	11.6 (0.788)	0.421 (0.039)	0.985 (0.001)	0.710 (0.007)
M1B: Δ = 0	84.4 (0.489)	36.2 (3.05)	50.0 (2.94)	13.7 (1.97)	5.53 (0.764)	0.420 (0.035)	0.985 (0.001)	0.710 (0.007)
M2: Δ = 2	70.0 (0.622)	29.2 (2.78)	42.2 (2.49)	17.3 (2.19)	4.58 (0.611)	0.409 (0.037)	0.963 (0.001)	0.698 (0.007)
M3: Δ = 4	31.5 (0.509)	10.9 (1.23)	21.3 (1.04)	4.53 (0.648)	9.10 (0.937)	0.339 (0.036)	0.841 (0.003)	0.638 (0.009)

1 $\sigma_{y}^{2}$ = phenotypic variance component on the measurement or ordinal scale; $\sigma_{u}^{2}$ = additive genetic variance; $\sigma_{e}^{2}$ = residual variance; $S_{u}^{2}$ = sample variance of estimated additive genetic values; $S_{e}^{2}$ = sample variance of residual effects; $h^{2}=\frac{\sigma_{u}^{2}}{\sigma_{u}^{2}+\sigma_{e}^{2}}$ = heritability.

2 $\rho_{y}$ = rank correlation between simulated phenotypes and the continuous or ordinal phenotypes used by each model; $\rho_{u}$ = rank correlation between simulated additive genetic values and estimated additive genetic values under each model.

3 M0 = model fitted on the original measurements as the phenotypes; M1A = model fitted on group means on the continuous scale as the phenotypes; M1B = model fitted on 5× 1–9 scores as the phenotypes without threshold shifts; M2 = model fitted on 5× 1–9 scores as the phenotypes after downscaling the thresholds by Δ = 2 in; M3 = model fitted on 5× 1–9 scores as the phenotypes after downscaling the thresholds by Δ = 4 in.

In what follows, we illustrate how to predict the impacts of phenotypic shifts using BLP. For M0: $\sigma_{y}^{2}$ = 22.7 cm^2^, $\sigma_{u}^{2}$ = 10.0 cm^2^, $\sigma_{e}^{2}$ = 13.1 cm^2^, and $h^{2}=\frac{10.0}{10.0+13.1}\approx$ 0.433 (Table 1). Using the exact category probabilities, the variance of the 1–9 scores is

$$\mathrm{Var}\left(s\right)=\sum_{j=1}^{9}j^{2}p_{j}-{\left(\sum_{j=1}^{9}jp_{j}\right)}^{2}=28.39-5^{2}\approx 3.39.$$

The BLP slope of 1–9 scores on genetic values (per cm) is

$$\beta =\frac{1}{\sigma_{y}}\sum_{j=1}^{k-1}\phi \left(\frac{\tau_{j}-\mu }{\sigma_{y}}\right)=\frac{1.82}{4.75}\approx 0.383.$$

The projected variances on the 1–9 scale are then given using Equations 11 and 12 as $\sigma_{u_{s}}^{2}=\beta^{2}\sigma_{u}^{2}=0.383^{2}\times 10.0\approx 1.47;$ $\sigma_{e_{s}}^{2}=\mathrm{Var}\left(s\right)-\beta^{2}\sigma_{u}^{2}=3.39-1.47\approx 1.92$ (Table 2).

**Table 2:** Projected variance components and heritability estimates using a best linear projection approach^1^

Δ	$\hat{\beta }$	1–9 scores			5× 1–9 scores			
		$\sigma_{s}^{2}$	$\sigma_{u}^{2}$	$\sigma_{e}^{2}$	$\sigma_{s}^{2}$	$\sigma_{u}^{2}$	$\sigma_{e}^{2}$	$h^{2}$
0 in (0 cm)	0.383	3.39	1.47	1.92	84.8	36.8	48.2	0.433
2 in (5.08 cm)	0.339	2.80	1.15	1.64	67.0	28.8	41.0	0.413
4 in (10.16 cm)	0.197	1.25	0.388	0.857	31.2	9.75	21.4	0.312

1 Δ = downscale offset of binning thresholds; $\hat{\beta }$ = regression coefficient; $\sigma_{s}^{2}$ = variance of 1–9 (or 5× 1–9) ordinal scores; $\sigma_{u}^{2}$ = projected additive genetic variance on the ordinal 1–9 (or 5× 1–9) scale; $\sigma_{e}^{2}$ = projected residual variance on the ordinal 1–9 (or 5× 1–9) scale; $h^{2}$ = heritability estimate.

According to Equations 19 to 21, rescaling the 1–9 scores to 5× (1–9) scores yields results comparable to those for M1B_Δ = 0 (Table 1): $\mathrm{Var}\left(5s\right)=5^{2}\times 3.39\approx 84.8;$ $\sigma_{u_{5s}}^{2}=5^{2}\times 1.47=36.8;$ $\sigma_{e_{5s}}^{2}=5^{2}\times 1.927=48.2.$ The projected heritability is

$$h_{5s}^{2}=\frac{36.8}{36.8+48.2}\approx 0.433.$$

Interestingly, with a correctly updated scoring system, the linear projection yielded comparable results to the linear model fitted on the original scale.

Now, downscale the thresholds by Δ = 2 in (5.08 cm; Table 2). The variance on the 1–9 scale is $\mathrm{Var}\left(s^{*}\right)=49.98-6.869^{2}\approx 2.80.$ The BLP slope becomes

$$\beta^{*}=\frac{1}{\sigma_{y}}\sum_{j=1}^{k-1}\phi \left(\frac{\tau_{j}-\mu -\delta }{\sigma_{y}}\right)=\frac{1.608}{4.75}\approx 0.339.$$

Projected genetic and residual variances are $\sigma_{u_{s*}}^{2}$ = $\beta^{*2}\sigma_{u}^{2}$ = 0.339^2^ × 10.0 ≈ 1.15 and $\sigma_{e_{s*}}^{2}$ = $\mathrm{Var}\left(s^{*}\right)$ − $\beta^{*2}\sigma_{u}^{2}$ = 2.80 − 1.15 ≈ 1.65.

On the rescaled 1–50 scale: $\mathrm{Var}\left(5s^{*}\right)=5^{2}\times \mathrm{Var}\left(s^{*}\right)=70.0,$ $\sigma_{u_{5s}}^{2}=5^{2}\times 1.154=28.8,$ $\sigma_{e_{5s*}}^{2}=5^{2}\times 1.64=41.0,$ and $h_{5s*}^{2}=\frac{28.8}{28.8+41.0}\approx 0.413.$

These values closely matched those for model 2 (Δ = 2; Table 1). Essentially, the reduced heritability estimate reflected the impact of a shift in ordinal phenotype distribution. The results for downscaling thresholds further by Δ = 4 in (10.16 cm) were obtained in a similar way, where $\sum_{j=1}^{k-1}\phi \left(\frac{\tau_{j}-\mu -\delta }{\sigma_{y}}\right)=0.934$ and $\beta^{*}=\frac{0.934}{4.75}\approx 0.197.$ Shifting the thresholds downward by 4 in further increases the mass in the upper bins, thereby reducing the BLP slope, genetic variance, and heritability (Table 2).

Finally, a reduction in heritability inevitably entails a loss of selection accuracy. Linearly back-transforming EBVs from the ordinal scale to the latent continuous scale, the expected accuracy of selection under a shifted system (Δ > 0) can be expressed as

[23] $$r\left(\Delta \right)=\sqrt{h_{s}^{2}\left(\Delta \right)}.$$

In our example, selection accuracy decreased from 0.659 (M0) to 0.650 (M1B: Δ = 0), 0.640 (M2: Δ = 2), and 0.582 (M3: Δ = 4). The rank correlations between simulated and estimated additive genetic values were slightly higher than *r*(Δ) but followed a similar decreasing trend: 0.717 (M0), 0.710 (Δ = 0), 0.698 (Δ = 2), and 0.638 (Δ = 4; Table 2).

For selecting the top-*α* animals, the expected response is

[24] $$R=i_{\alpha }r\left(\Delta \right)\sigma_{u},$$

where *i_α_* is the selection intensity and *σ_u_* is the additive genetic standard deviation. For example, when selecting the top 5% (*α* = 0.05) of animals, iα=ϕz1-αϕz1-ααα with *z*_0.95_ = 1.645, which gives *i*_0.05_ = 2.063. The expected selection responses (in the same unit as the latent scale) are 1.35 (SD = 4.29) under Δ = 0, 1.32 (SD = 4.19) under Δ = 2, and 1.15 (SD = 3.65) under Δ = 4.

In addition to overall accuracy, we also assessed accuracy for specific selection objectives—selecting the tallest, intermediate, and smallest animals—under selection intensities ranging from 5% to 50%. The relative selection accuracy (**RSA**) was defined as the ratio of rank correlation between the estimated additive genetic values obtained from the ordinal-scale evaluation and those on the continuous-scale baseline model (M0). This metric reflects a realistic breeding scenario in which actual breeding values are unknown, and evaluations based on observed phenotypes serve as the benchmark, against which alternative scoring systems are compared.

Across 10 replicates, selecting the top 5% of animals based on outdated scores (Δ = 2) resulted in an average RSA reduction of 28.5% (Figure 1). This substantial loss in selection accuracy stemmed from misclassifying nonelite animals as “very tall” due to score compression and misaligned bin boundaries, which concentrated scores in the highest categories (see also the bottom-left panel of the Graphical Abstract). Nevertheless, the adverse effect diminished as selection intensity broadened: the RSA loss declined from 28.5% to 16.0% and then to only 0.6% as selection proportion increased from 5% to 10% and then to 50%. This dilution effect arises because a broader selection included animals less affected by threshold misalignment. Hence, outdated scoring thresholds can systematically erode both selection accuracy and expected genetic gain, with the greatest impact observed under intense selection for extreme phenotypes.

**Figure 1:**
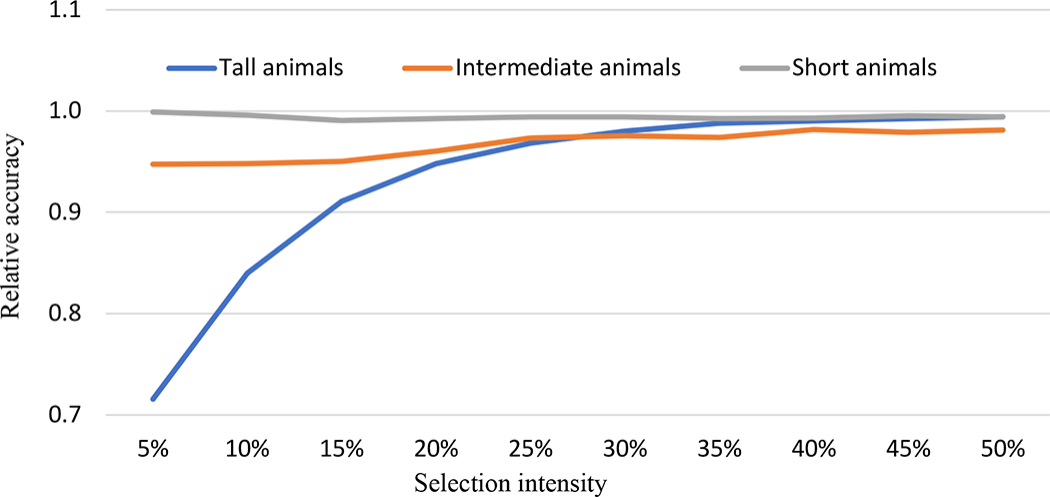
Selection accuracy using 5× 1–9 ordinal scores as phenotypes under 3 selection scenarios, relative to the selection accuracy using original stature measurements as phenotypes.

In conclusion, phenotypic shifts have tangible and significant consequences for genetic evaluations and selection outcomes. Outdated scoring systems distort the mapping between biological measurements and categorical scores, leading to biased variance components, underestimated heritability, and reduced selection accuracy. Therefore, periodic recalibration of threshold boundaries is essential to keep the ordinal scoring systems aligned with current population distributions and to safeguard the validity of genetic improvement programs.

While stature provides a clear example of scale drift in an ordinal scoring system, other traits recorded on discrete scales—such as BCS—may also be susceptible to similar distributional shifts over time. A systematic investigation of these traits could help determine whether recalibration is needed more broadly across fitness and welfare-related phenotypes.
